# Efetividade e Segurança da Remoção de Cabos-Eletrodos Transvenosos de Marca-Passos e Desfibriladores Implantáveis no Cenário da Prática Clínica Real

**DOI:** 10.36660/abc.20200476

**Published:** 2020-12-01

**Authors:** Roberto Costa, Katia Regina da Silva, Elizabeth Sartori Crevelari, Wagner Tadeu Jurevicius Nascimento, Marcia Mitie Nagumo, Martino Martinelli, Fabio Biscegli Jatene

**Affiliations:** 1 Hospital das Clínicas Faculdade de Medicina Universidade de São Paulo São PauloSP Brasil Instituto do Coração do Hospital das Clínicas da Faculdade de Medicina da Universidade de São Paulo, São Paulo, SP – Brasil

**Keywords:** Eletrodos Implantados, Marca-Passo, Estimulação Cardíaca Artificial, Extração de Cabos-eletrodos, Infecção, Efetividade, Complicações Cirúrgicas, Mortalidade

## Abstract

**Fundamento:**

Remoção de cabos-eletrodos de dispositivos cardíacos eletrônicos implantáveis (DCEI) é procedimento pouco frequente e sua realização exige longo treinamento profissional e infraestrutura adequada.

**Objetivos:**

Avaliar a efetividade e a segurança da remoção de cabos-eletrodos de DCEI e determinar fatores de risco para complicações cirúrgicas e mortalidade em 30 dias.

**Métodos:**

Estudo prospectivo com dados derivados da prática clínica. De janeiro/2014 a abril/2020, foram incluídos, consecutivamente, 365 pacientes submetidos à remoção de cabos-eletrodos, independentemente da indicação e técnica cirúrgica utilizada. Os desfechos primários foram: taxa de sucesso do procedimento, taxa combinada de complicações maiores e morte intraoperatória. Os desfechos secundários foram: fatores de risco para complicações intraoperatórias maiores e morte em 30 dias. Empregou-se análise univariada e multivariada, com nível de significância de 5%.

**Resultados:**

A taxa de sucesso do procedimento foi de 96,7%, sendo 90,1% de sucesso completo e 6,6% de sucesso clínico. Complicações maiores intraoperatórias ocorreram em 15 (4,1%) pacientes. Fatores preditores de complicações maiores foram: tempo de implante dos cabos-eletrodos ≥ 7 anos (OR= 3,78, p= 0,046) e mudança de estratégia cirúrgica (OR= 5,30, p= 0,023). Classe funcional III-IV (OR= 6,98, p<0,001), insuficiência renal (OR= 5,75, p=0,001), infecção no DCEI (OR= 13,30, p<0,001), número de procedimentos realizados (OR= 77,32, p<0,001) e complicações maiores intraoperatórias (OR= 38,84, p<0,001) foram fatores preditores para mortalidade em 30 dias.

**Conclusões:**

Os resultados desse estudo, que é o maior registro prospectivo de remoção de cabos-eletrodos da América Latina, confirmam a segurança e a efetividade desse procedimento no cenário da prática clínica real. (Arq Bras Cardiol. 2020; 115(6):1114-1124)

## Introdução

A remoção de cabos-eletrodos de dispositivos cardíacos eletrônicos implantáveis (DCEI) é procedimento pouco frequente e sua realização exige longo treinamento profissional e infraestrutura adequada.^1 - 5^ Embora as indicações estejam bem estabelecidas em diretrizes médicas, sua utilização varia de acordo com a expertise de cada centro, sendo usada quase que exclusivamente para o tratamento de processos infecciosos relacionados aos DCEIs nos serviços menos especializados e em maior escala em serviços com maior experiência.^2 - 9^

Como a maioria dos dispositivos implantados utiliza o acesso venoso para o implante dos cabos-eletrodos, as técnicas transvenosas são as mais utilizadas. Na atualidade, a abertura do tórax é pouco empregada para a remoção de cabos-eletrodos, sendo utilizada quase que exclusivamente para a remoção de cabos-eletrodos epicárdicos. Para eletrodos transvenosos, a cirurgia aberta está restrita aos casos em que existem grandes vegetações próximas aos cabos-eletrodos ou para a correção de complicações em extrações transvenosas.^3^

Aderências dos cabos-eletrodos ao miocárdio, às veias ou entre os próprios cabos são muito frequentes. Vários fatores estão relacionados à formação de aderências como: o tempo de implante, o tipo de cabo-eletrodo, o número de cabos implantados, a idade e o sexo do paciente.^6^ Para desfazer essas aderências, ferramentas específicas têm sido empregadas, cada uma com suas indicações, vantagens e desvantagens.^3 , 7^ Em uma visão geral, as taxas de sucesso da remoção reportadas variam de 90 a 98%.^1 , 7 , 10 , 11^ A despeito dos resultados satisfatórios obtidos, complicações catastróficas podem ocorrer durante a extração e, quando ocorrem, procedimentos cirúrgicos a céu aberto podem ser necessários para a correção.^9 , 12 , 13^ Complicações graves são reportadas em 1 a 10% e morte intraoperatória em 0,2 a 5,7% dos casos. A mortalidade total nos primeiros 30 dias de pós-operatório varia de 2,1 a 10% dos casos.^10 - 13^

A efetividade das ferramentas utilizadas para extração e os fatores de risco associados à ocorrência de complicações catastróficas ainda não estão bem estabelecidos na literatura.^1 , 2 , 10 - 14^As baixas taxas de utilização e de complicações, associadas à diversidade de técnicas e de ferramentas disponíveis para extração, dificultam essa comparação. No cenário nacional, a falta de incorporação de recursos tecnológicos específicos para extração no Sistema Único de Saúde (SUS) justifica a limitação do seu uso, bem como a inexistência de dados confiáveis resultantes da extração de cabos-eletrodos no Brasil.

A finalidade do presente estudo foi avaliar a efetividade e a segurança da remoção de cabos-eletrodos de marca-passos e desfibriladores, visando determinar a taxa de sucesso dos procedimentos, a taxa de complicações cirúrgicas, a mortalidade operatória e a mortalidade total nos primeiros 30 dias após a alta hospitalar, assim como os fatores de risco para desfechos desfavoráveis.

## Métodos

### Desenho e local do estudo

Trata-se de um estudo prospectivo observacional com dados derivados da prática clínica. Os dados foram coletados em dois momentos distintos: (1) na internação hospitalar índice, ou seja, no episódio hospitalar relacionado ao procedimento de remoção dos cabos-eletrodos; (2) trinta dias após a alta hospitalar.

Esse estudo foi realizado em um hospital cardiológico de alta complexidade, tendo sido aprovado pelo Comitê de Ética em Pesquisa da Instituição.

### População do estudo

De janeiro de 2014 a abril de 2020, foram incluídos, consecutivamente, todos os pacientes submetidos à remoção de cabos-eletrodos, independentemente da indicação cirúrgica e da técnica utilizada. Não foram incluídos indivíduos cuja remoção foi feita em função de transplante cardíaco ortotópico.

### Desfechos do estudo

Os desfechos primários do estudo foram: a efetividade do procedimento, expressa pela taxa de sucesso clínico, e a sua segurança, expressa pela taxa combinada de complicações maiores e morte intraoperatória.

Foram utilizadas as definições das diretrizes europeias.^8^ Considerou-se sucesso, no presente estudo, a remoção de todo o material do cabo-eletrodo alvo da extração ou a retenção de até 4 cm do cabo-eletrodo, desde que a extração não fosse realizada para tratamento de infecção no DCEI. No sentido oposto, foi considerada falha do procedimento a retenção de fragmento maior do que 4cm; a retenção de fragmento de cabo-eletrodo de qualquer tamanho em paciente com infecção no DCEI; a ocorrência de qualquer complicação incapacitante permanente ou morte intraoperatória relacionada ao procedimento.

Os desfechos secundários foram fatores de risco para complicações intraoperatórias maiores e para a morte nos primeiros 30 dias após a alta hospitalar.

### Etapas do estudo

#### Avaliação pré-operatória

Pacientes com indicação de remoção de cabos-eletrodos e que preenchiam os critérios de elegibilidade do estudo, foram submetidos à avaliação pré-operatória de rotina, constituída por avaliação clínica, laboratorial e exames de imagem.

Conforme rotina institucional, os pacientes foram submetidos a: radiografia do tórax, para determinação da posição dos cabos-eletrodos; venografia digital bilateral para estudo do território venoso e, quando havia diagnóstico de infecção no DCEI, ecocardiograma transesofágico.

#### Procedimento cirúrgico

Os procedimentos foram realizados sob anestesia geral com intubação orotraqueal, monitoramento completo, inclusive com ecocardiograma transesofágico.

As operações foram agrupadas pela via de acesso dos cabos-eletrodos em: (1) Cabos-eletrodos epicárdicos, removidos por cirurgia aberta; (2) Cabos-eletrodos transvenosos, removidos, preferencialmente, por técnica transvenosa.

#### Remoção pela técnica de acesso pela entrada do cabo-eletrodo na veia

Cabos-eletrodos passíveis de manipulação extravascular foram removidos por abordagem sequencial que se iniciava pela tração direta simples e passava para a extração transvenosa com o uso de ferramentas no caso de insucesso da tração direta.

- Remoção por tração simples: a tentativa de remover o(s) cabo(s)-eletrodo(s) sem o uso de ferramentas específicas para extração foi feita em todos os casos. Com esse objetivo, um guia convencional de cabo-eletrodo era passado no lúmen do cabo a ser removido, sendo aplicada tração firme e contínua, na tentativa de liberar o cabo-eletrodo do miocárdio e de seu trajeto nas veias. No caso de insucesso dessa manobra, o próximo passo foi o uso de ferramentas de extração.
- Extração transvenosa: o procedimento de extração foi realizado com uso de guia de travamento ( *locking stylet* ). A dissecção intravascular foi feita com a ferramenta disponível para o caso em questão (bainha energizada por raios laser, bainha mecânica não energizada ou bainha mecânica rotacional). O objetivo da dissecção era conduzir a bainha até o local de implante do cabo-eletrodo no miocárdio para realização da contra-tração.

#### Remoção por captura intravascular

Nos casos em que o cabo-eletrodo não estava acessível para manipulação extravascular (“cabo flutuante”) foi realizada a captura intravascular por acesso femoral ou jugular. Após a captura intravascular, a liberação da ponta do cabo-eletrodo foi feita por tração simples ou por contra-tração, dependendo da necessidade de cada caso.

#### Mudança de estratégia cirúrgica

Nos casos em que não se conseguiu avançar sobre o cabo-eletrodo com a ferramenta utilizada como primeira opção, pode ter sido necessária a mudança de estratégia, incluindo o uso de outro tipo de ferramenta, captura intravascular ou cirurgia aberta.

#### Reimplante do DCEI

Nos pacientes em que não havia o diagnóstico de infecção no DCEI, o implante do novo dispositivo foi realizado no mesmo ato cirúrgico. Quando havia infecção no DCEI, o novo implante foi realizado sempre em outra operação, após o controle da infecção.

## Avaliação pós-operatória e seguimento clínico

No momento da alta hospitalar foram coletados dados do pós-operatório imediato, priorizando-se a pesquisa de complicações perioperatórias.

De acordo com a rotina habitual, todos os pacientes foram avaliados em regime ambulatorial, 30 dias após a alta hospitalar. Essa avaliação priorizou a pesquisa de complicações decorrentes do procedimento, a necessidade de readmissão hospitalar ou de reintervenção cirúrgica.

## Coleta eletrônica e gerenciamento dos dados

**Figura 1:**
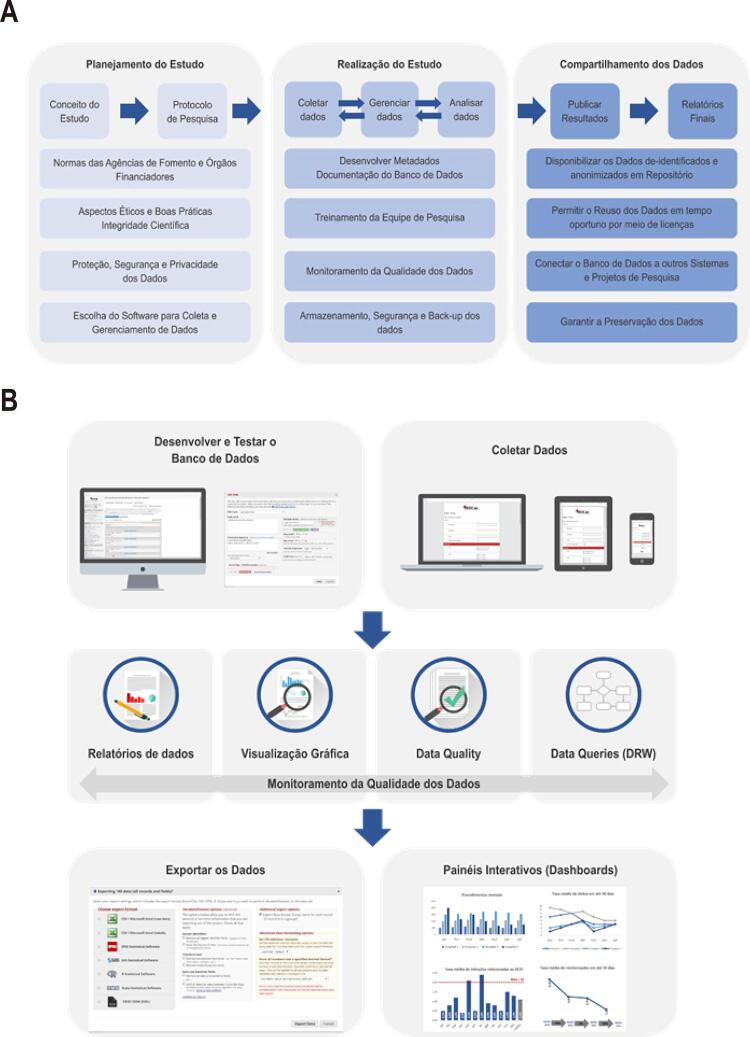

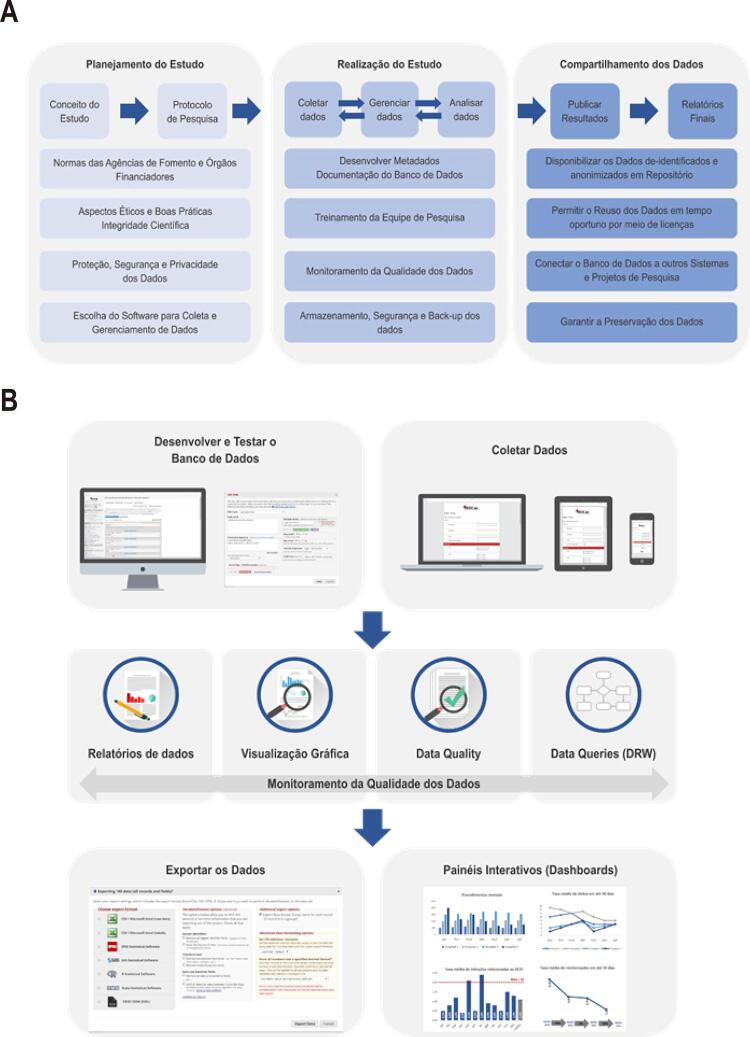
– Coleta eletrônica e gerenciamento dos dados do estudo. A) Etapas do gerenciamento de dados. B) Funcionalidades do REDCap utilizadas no estudo.

Para assegurar a qualidade do banco de dados, adotamos infraestrutura padronizada anteriormente,^15^ que contemplou: (1) Plano de gerenciamento de dados; (2) Definição da terminologia dos elementos de dados; (3) Desenvolvimento de formulários eletrônicos no sistema REDCap;^16^ (4) Parametrização de funcionalidades específicas do REDCap; (5) Treinamento da equipe de coleta de dados; (6) Monitoramento dinâmico da qualidade dos dados; (7) Integração do sistema REDCap com ferramenta de *Business Intelligence* , para criação de painéis interativos dinâmicos ( *dashboards* ), permitindo a visualização dos resultados em tempo real, em ambiente tecnológico hospedado em nuvens. Para favorecer a reprodutibilidade do estudo e a divulgação de resultados anonimizados e de-identificados em tempo real, optou-se pela plataforma *Open Source* ( *Shinydashboard* , RStudio) ( Figura 1 ).

## Variáveis estudadas e análise estatística

Foram analisadas como variáveis independentes para os desfechos: dados demográficos, dados clínicos basais, características do DCEI removido, tipo de remoção e uso de ferramentas específicas para extração.

Empregou-se análise univariada para a pesquisa de fatores de risco associados aos desfechos, adotando-se o nível de significância de 5%. Foi empregado o modelo de regressão logística multivariada com o método *stepwise* de seleção de variáveis para a pesquisa de fatores de risco independentes, utilizando-se como critério de inclusão as associações que apresentaram p≤ 0,10 na análise univariada.

## Resultados

### Características basais

No período do estudo, foram incluídos 365 pacientes submetidos de um a três procedimentos para remoção de cabos-eletrodos até o final de seu tratamento. Houve predomínio de indivíduos do sexo masculino (55,6%) e a idade média foi de 59,8 ± 19,3 anos, com mediana de 63,0 anos ( Tabela 1 ).

**Tabela 1:** – Dados demográficos e clínicos basais

Características Basais	
Sexo masculino, n (%)	203 (55,6)
Idade (anos), média ± DP (variação)	59,8 ± 19,3 (0,8 - 98,0)
Raça branca, n (%)	306 (83,8)
**Classe Funcional (NYHA), n (%)**	
I – II	314 (86,0)
III - IV	51 (14,0)
**Doença cardíaca estrutural, n (%)**	
Distúrbios da condução e do ritmo (sem doença cardíaca estrutural)	153 (41,9)
Doença de Chagas	68 (18,6)
Cardiopatia isquêmica	46 (12,6)
Cardiopatia não-isquêmica	86 (23,6)
Defeito cardíaco congênito	12 (3,3)
**Comorbidades, n (%)**	
Hipertensão arterial	186 (51,0)
Diabetes	60 (16,4)
Fibrilação atrial	70 (19,2)
Insuficiência cardíaca	140 (38,4)
Insuficiência renal crônica	43 (11,8)
Acidente vascular cerebral	33 (9,0)
Neoplasia em tratamento atual ou recente	9 (2,5)
Fração de ejeção do ventrículo esquerdo, média ± DP (variação)	49,1 ± 16,3 (15- 82,0)
**Tipo de DCEI em uso, n (%)**	
MP unicameral	41 (11,2)
MP dupla câmara	170 (46,6)
CDI unicameral	33 (9,0)
CDI dupla câmara	64 (17,5)
TRC-MP	24 (6,6)
TRC-CDI	33 (9,0)
**Indicação da remoção de cabos-eletrodos, n (%)**	
Tratamento de infecção	104 (28,5)
Superpopulação de cabos-eletrodos	207 (56,7)
Obtenção de acesso venoso	22 (6,0)
Deslocamento de cabos-eletrodos	26 (7,1)
Tratamento de complicações tromboembólicas	3 (0,8)
Outros problemas	3 (0,8)
**Diagnóstico principal, n (%)**	
Infecção na loja do gerador de pulsos	57 (15,6)
Infecção intravascular sem vegetação	10 (2,7)
Infecção intravascular com vegetação	37 (10,1)
Disfunção de cabos-eletrodos	218 (59,7)
Necessidade de mudança do modo de estimulação	35 (9,6)
Outros	8 (2,2)

*CDI: cardioversor-desfibrilador implantável; DP: desvio padrão; MP: marca-passo; NYHA: New York Heart Association; TRC: terapia de ressincronização cardíaca.*

A maioria dos indivíduos era oligossintomática para insuficiência cardíaca (86,0%), não havendo doença cardíaca estrutural em 39,1% deles. Os dispositivos previamente implantados eram marca-passos em 57,8% e cardioversores-desfibriladores implantáveis (CDI) em 33,1% dos casos.

O principal motivo para a realização do procedimento foi a disfunção de cabos-eletrodos em 218 (59,7%) pacientes. Infecção no DCEI, com ou sem vegetações intracardíacas, foi a causa da remoção em 104 (28,5%) casos.

### Características operatórias

Nos 365 pacientes incluídos foram realizados 378 procedimentos de remoção de cabos-eletrodos. Em 9 (2,5%) casos, mais do que um procedimento foi necessário. Foram removidos 634 cabos-eletrodos, 521 de marca-passo e 113 de CDI. O tempo médio de permanência dos cabos-eletrodos era 7,5 ± 6,6 anos, com máximo de 39 anos ( Tabela 2 ).

**Tabela 2:** – Dados dos procedimentos de remoção de cabos-eletrodos

Características Operatórias	
Duração do procedimento (minutos), média ± DP (variação)	147,9 ± 82,0 (20 - 635)
**Número total de cabos-eletrodos removidos por paciente, n (%)**	
Um	197 (54,0)
Dois	125 (34,2)
Três	32 (8,8)
Quatro	11 (3,0)
Tempo de permanência dos cabos-eletrodos (anos), média ± DP (variação)	7,5 ± 6,6 (0 – 39)
**Local de implante dos cabos-eletrodos, n (%)**	
Atrial	221 (34,9)
Ventrículo direito (marca-passo)	258 (40,7)
Ventrículo direito (CDI)	113 (17,8)
Seio coronário	42 (6,6)
**Técnica operatória principal, n (%)**	
Tração simples	140 (38,4)
Tração simples com dilatação	22 (6,0)
Extração transvenosa com acesso pela entrada do eletrodo na veia	180 (49,3)
Toracotomia sem circulação extracorpórea	17 (4,7)
Toracotomia com circulação extracorpórea	6 (1,6)
**Recursos tecnológicos específicos, n (%)**	
Guia de travamento	183 (50,1)
Bainha mecânica não energizada	77 (21,1)
Bainha mecânica rotacional	23 (6,3)
Bainha energizada por raios laser	80 (21,9)
**Mudança na estratégia cirúrgica, n (%)**	
Remoção por tração simples para extração transvenosa	2 (0,5)
Remoção por tração simples para cirurgia aberta	2 (0,5)
Mudança do tipo de ferramenta de dissecção intravascular	6 (1,6)
Extração pela entrada do eletrodo na veia para captura intravascular	5 (1,4)
Extração pela entrada do eletrodo na veia para cirurgia aberta	5 (1,4)
Múltiplas	1 (0,3)

*CDI: cardioversor-desfibrilador implantável; DP: desvio padrão.*

Cirurgia aberta para a remoção de cabos epicárdicos foi realizada em 17(4,6%) pacientes. Cirurgia com circulação extracorpórea (CEC) foi necessária como primeira abordagem em 6 (1,6%) e, como mudança de estratégia, em 7 (1,9%) casos. Em todos os procedimentos realizados pela entrada da veia, a tração simples foi tentada, obtendo-se sucesso em 140 (38,4%) pacientes. Nos demais, foi realizada extração transvenosa. Guia de travamento foi usada em 183 casos, bainha energizada por raios laser em 80 (21,9%), bainha mecânica não energizada em 77 (21,1%), e bainha mecânica rotacional em 23 (6,3%) pacientes.

Mudança de estratégia foi necessária no mesmo ato cirúrgico em 12 (3,3%) pacientes e, como procedimento independente, em 9 (2,5%). A mudança de estratégia exigiu o uso de outro tipo de ferramenta de dissecção em 6 (1,6%), captura intravascular em 5 (1,4%), ou cirurgia aberta com CEC em 7 (1,9%) casos.

### Desfechos Primários do Estudo

#### Efetividade do procedimento

A taxa de sucesso da remoção de cabos-eletrodos foi de 96,7% (IC 95%= 94,5 – 98,5%), com sucesso completo em 329 (90,1%, IC 95%= 87,1 – 93,2%) pacientes e sucesso clínico em 24 (6,6%, IC 95%= 4,1 – 9,1%).

A taxa de falha do procedimento de extração foi de 3,2% (IC 95%= 1,5 – 5,1%). A falha foi decorrente de retenção de fragmento >4cm em 3 (0,8%) pacientes sem infecção, retenção de qualquer fragmento em 7 (1,9%) pacientes com infecção no DCEI e complicação maior que necessitou correção cirúrgica em 2 (0,5%) pacientes.

#### Segurança do procedimento

O desfecho composto pelas complicações maiores e morte intraoperatória ocorreu em 15 (4,1%, IC 95%= 2,1 – 6,1) pacientes. Óbito intraoperatório ocorreu em apenas 1 (0,3%) caso em decorrência de avulsão de estruturas cardíacas, ocasionando choque hemorrágico. Apenas 2 (0,5%) pacientes apresentaram mais do que uma complicação maior simultaneamente. Complicações menores intraoperatórias foram observadas em 10 (2,7%) pacientes, sendo que em apenas um caso houve complicações menores concomitantes ( Tabela 3 ).

**Tabela 3:** – Complicações maiores e menores intraoperatórias relacionadas à remoção de cabos-eletrodos

Complicações Intraoperatórias	
Complicações maiores, n (%)	
Óbito	1 (0,3)
Parada cardiorrespiratória	6 (1,6)
Arritmia instável	5 (1,4)
Tamponamento cardíaco	3 (0,8)
Avulsão de estruturas cardíacas	2 (0,5)
Sangramento instável	3 (0,8)
**Complicações menores, n (%)**	
Hemotórax com necessidade de drenagem	2 (0,5)
Pneumotórax com necessidade de drenagem	2 (0,5)
Pneumotórax mínimo, sem necessidade de drenagem	1 (0,3)
Derrame pericárdico	4 (1,1)
Sangramento estável	4 (1,1)
Desposicionamento de cabos-eletrodos	2 (0,5)

#### Eventos pós-operatórios

A mediana do tempo de internação dos pacientes foi de 9 dias, variando de 1 a 169 dias. O principal motivo para longa permanência hospitalar foi a necessidade de antibioticoterapia prolongada em pacientes com infecção no DCEI. Após a alta hospitalar, 13 (3,6%) pacientes apresentaram complicações e 8 (2,2%) foram novamente operados. ( Tabela 4 ).

**Tabela 4:** – Eventos pós-operatórios após remoção de cabos-eletrodos

Eventos pós-operatórios	
Tempo de permanência hospitalar (dias), média ± DP (variação)	17,4 ± 21,6 (1- 169)
**Complicações maiores na fase hospitalar**	
Óbitos	29 (7,9)
Parada cardiorrespiratória	3 (0,8)
Arritmia instável	1 (0,3)
Tamponamento cardíaco	1 (0,3)
Embolia pulmonar	2 (0,5)
Sangramento instável	2 (0,5)
Sepse	13 (3,6)
**Complicações menores na fase hospitalar**	
Hemotórax com necessidade de drenagem	1 (0,3)
Pneumotórax com necessidade de drenagem	2 (0,5)
Hematoma de loja	6 (1,6)
Desposicionamento de cabos-eletrodos	2 (0,5)
**Complicações ocorridas 30 dias após a alta**	
Óbitos	4 (1,1)
Readmissão hospitalar	33 (9,0)
Reoperação relacionada ao DCEI	8 (2,2)
Infecção relacionada ao DCEI	1 (0,3)
Disfunção em cabo-eletrodo	3 (0,8)
Embolia pulmonar	2 (0,5)
Trombose venosa profunda no membro superior ipsilateral	7 (1,9)

*DP: desvio padrão; DCEI: dispositivos cardíacos eletrônicos implantáveis.*

A mortalidade total foi de 34 (9,3%) casos, sendo 1 (0,3%) óbito intraoperatório, 29 (7,9%) no período pós-operatório hospitalar e 4 (1,1%) após a alta hospitalar. A causa mais frequente de morte foi a infecção no DCEI, em 20 (5,5%) casos, seguida por causas cardiovasculares em 6 (1,6%), complicações do procedimento de extração em 5 (1,4%) e causas não cardiovasculares em 3 (0,8%) pacientes.

#### Fatores de risco para complicações maiores intraoperatórias e mortalidade

De acordo com a análise univariada, o tempo de implante dos cabos-eletrodos (p= 0,015) e a mudança na estratégia cirúrgica (p= 0,016) estiveram associados com a maior ocorrência de complicações maiores intraoperatórias.

Idade (p= 0,004), classe funcional (p<0,001), insuficiência cardíaca (p= 0,003), insuficiência renal (p<0,001), infecção relacionada ao DCEI (P<0,001), total de cabos-eletrodos removidos (p<0,001), tipo de cabo-eletrodo removido (p=0,029), número total de procedimentos (p<0,001), resultados do procedimento (p=0,002), mudança na estratégia cirúrgica (p=0,005) e complicações maiores intraoperatórias (p<0,001) foram fatores associados à mortalidade total de 30 dias ( Tabela 5 ).

**Tabela 5:** – Fatores de risco para complicações maiores intraoperatórias e mortalidade total em 30 dias

Características	Sem complicações (n= 350)	Com complicações (n= 15)	p	Óbito ausente (n= 331)	Óbito presente (n= 34)	p
Sexo masculino, n (%)	197 (56,3)	9 (60,0)	0,214	185 (55,9)	18 (52,9)	0,741
Idade (anos), média ± DP	59,9 ± 19,2	57,7 ± 22,5	0,670	58,8 ± 19.4	69,0 ± 15.9	0,004
**Classe Funcional (NYHA), n (%)**						
I – II	303 (86,6)	11 (73,4)	0,142	292 (88,2)	22 (64,7)	
III – IV	47 (13,4)	4 (26,6)		39 (11,8)	12 (35,3)	<0,001
**Doença cardíaca, n (%)**						
Sem doença cardíaca estrutural	147 (42,0)	6 (40,0)		142 (42,9)	11 (32,3)	
Cardiopatia isquêmica	46 (13,1)	0	0,258	42 (12,7)	4 (11,8)	0,420
Cardiopatia não-isquêmica	157 (44,9)	9 (60,0)		147 (44,4)	19 (55,9)	
**Comorbidades, n (%)**						
Diabetes (ausente)	290 (82,9)	15 (100)		278 (84,0)	27 (79,4)	
Diabetes (presente)	60 (17,1)	0	0,145	53 (16,0)	7 (20,6)	0,493
Insuficiência cardíaca (ausente)	217 (62,0)	8 (53,3)		212 (64,1)	13 (38,2)	
Insuficiência cardíaca (presente)	133 (38,0)	7 (46,7)	0,499	119 (35,9)	21 (61,8)	0,003
Insuficiência renal crônica (ausente)	307 (87,7)	15 (100)		299 (90,3)	23 (67,6)	
Insuficiência renal crônica (presente)	43 (12,3)	0	0,233	32 (9,7)	11 (32,4)	<0,001
**Tipo de DCEI, n (%)**						
Unicameral	71 (20,3)	3 (20,0)		66 (19,9)	8 (23,5)	
Dupla câmara	225 (64,3)	9 (60,0)	0,862	216 (65,3)	18 (53,0)	0,296
Ressincronizador cardíaco	54 (15,4)	3 (20,0)		49 (14,8)	8 (23,5)	
**Indicação da remoção, n (%)**						
Causas não infecciosas	250 (71,4)	11 (73,3)		253 (76,4)	8 (23,5)	
Infecção no DCEI	100 (28,6)	4 (26,7)	1,000	78 (23,6)	26 (76,5)	<0,001
**Número de cabos-eletrodos removidos, n (%)**						
1 - 2	310 (88,6)	12 (80,0)		301 (90,9)	21 (61,8)	
3 - 4	40 (11,4)	3 (20,0)	0,400	30 (9,1)	13 (38,2)	<0,001
Tempo de implante dos cabos-eletrodos, n (%)	7,3 ± 6,6	11,2 ± 6,7	0,015	7,1 ± 6,2	10,1 ± 9,5	0,142
**Tipo de cabo-eletrodo, n (%)**						
Marca-passo	252 (72,0)	12 (80,0)		234 (70,7)	30 (8,2)	
CDI	98 (28,0)	3 (20,0)	0,768	97 (29,3)	4 (11,8)	0,029
**Técnica operatória, n (%)**						
Tração simples com ou sem dilatação	159 (45,4)	3 (20,0)		148 (44,7)	14 (41,2)	
Extração transvenosa	169 (48,3)	11 (73,3)	0,140	164 (49,6)	16 (47,1)	0,386
Cirurgia aberta	22 (6,3)	1 (6,7)		19 (5,7)	4 (11,7)	
**Mudança na estratégia cirúrgica, n (%)**						
Não	339 (96,8)	12 (80,0)		322 (97,3)	29 (85,3)	
Sim	11 (3,2)	3 (30,0)	0,016	9 (2,7)	5 (14,7)	0,005
**Número de procedimentos realizados, n (%)**						
Um	342 (97,7)	14 (93,3)		328 (99,1)	28 (82,4)	
Dois	5 (1,4)	1 (6,7)	0,317	3 (0,9)	3 (8,8)	<0,001
Três	3 (0,9)	0		0	3 (8,8)	
**Resultados do procedimento, n (%)**						
Sucesso	340 (97,1)	13 (86,7)		324 (97,9)	29 (85,3)	
Insucesso	10 (2,9)	2 (13,3)	0,082	7 (2,1)	5 (14,7)	0,002
**Complicações maiores intraoperatórias, n (%)**						
Ausente	-	-		323 (97,6)	27 (79,4)	
Presente	-	-		8 (2,4)	7 (20,6)	<0,001

*DP: desvio padrão; NYHA: New York Heart Association; DCEI: dispositivos cardíacos eletrônicos implantáveis; CDI: cardioversor-desfibrilador implantável.*

Pela análise multivariada, foi possível identificar que o tempo de implante dos cabos-eletrodos ≥ 7 anos e a mudança de estratégia cirúrgica foram fatores preditores independentes para a ocorrência de complicações maiores intraoperatórias. Classe funcional III-IV, insuficiência renal crônica, infecção no DCEI, número de procedimentos realizados e complicações maiores intraoperatórias foram fatores independentes para mortalidade total em até 30 dias ( Tabela 6 ).

**Tabela 6:** – Preditores independentes de complicações maiores intraoperatórias e mortalidade total

Preditores independentes	Odds ratio	IC 95%		P
Complicações maiores intraoperatórias				
Tempo de implante dos cabos-eletrodos ≥ 7 anos	3,78	1,02	13,95	0,046
Mudança de estratégia cirúrgica	5,30	1,26	22,22	0,023
**Mortalidade**				
Classe funcional (NYHA) III - IV	6,98	2,45	19,86	<0,001
Insuficiência renal crônica	5,75	1,98	16,67	0,001
Infecção no DCEI	13,30	4,45	39,69	<0,001
Número total de procedimentos	77,32	8,64	692,19	<0,001
Complicações maiores intraoperatórias	38,84	7,83	192,77	<0,001
Remoção de eletrodo de CDI	0,22	0,06	0,81	0,023

*NYHA: New York Heart Association; DCEI: dispositivos cardíacos eletrônicos implantáveis; CDI: cardioversor-desfibrilador implantável.*

## Discussão

O presente estudo é o primeiro registro da América Latina projetado para avaliar, prospectivamente, dados sistematizados de procedimentos de remoção de cabos-eletrodos no cenário da prática clínica real. Dessa forma, nesta amostra composta por 634 cabos-eletrodos removidos de 365 pacientes seguidos por 30 dias após a alta hospitalar, estão representados pacientes de todas as idades, com diferentes cardiopatias estruturais e comorbidades, assim como todos os tipos de DCEI e cabos-eletrodos.

A observação de que a infecção no DCEI não foi a principal indicação de remoção de cabos-eletrodos difere da maioria dos estudos, em que mais da metade da população é portadora de infecção.^3 , 7 , 11 , 12^A alta prevalência de indivíduos não infectados foi fundamental para que os objetivos do estudo fossem atingidos, por serem pacientes menos idosos e com grande tempo de permanência dos cabos-eletrodos. Essas características aumentaram a representatividade das complicações associadas ao próprio procedimento e de seu impacto na mortalidade, diminuindo, relativamente, os efeitos da infecção, das comorbidades e outras características inerentes ao próprio paciente.

O desenvolvimento de ferramentas específicas para extração transvenosa tem sido fundamental para garantir maior segurança aos pacientes. A comparação da segurança e da efetividade destas ferramentas, contudo, é problemática, pois muitas delas são utilizadas como soluções de *backup* para casos difíceis ou para corrigir complicações.^8 , 12 - 14^ No presente estudo, foram utilizados os principais tipos de ferramentas disponíveis para extração transvenosa. A falta da incorporação da extração transvenosa no SUS, entretanto, impediu a adequada comparação das diversas tecnologias disponíveis, uma vez que a escolha da ferramenta foi definida por sua disponibilidade para cada caso. A despeito desse viés, guia de travamento foi usada em 50,1% dos casos, bainhas energizadas por raios laser em 21,9%, bainhas mecânicas não energizadas em 21,1%, e bainhas mecânicas rotacionais em 6,3% dos pacientes.

Dessa forma, a segurança e a efetividade da extração de cabos-eletrodos, desfechos primários do estudo, puderam ser avaliados de forma robusta. A taxa de sucesso de 96,7%, assim como a taxa de complicações maiores de 4,1%, foram comparáveis às dos serviços internacionais com grande volume de extrações.^7 , 12 , 14 , 17 , 18^ A mortalidade total de 9,3% foi decorrente, principalmente, de causas relacionadas ao paciente e, em apenas 1,4%, por complicações da remoção.

O risco de complicações catastróficas tem sido o principal obstáculo para a indicação da extração de eletrodos nos casos não obrigatórios. Esse fato tem motivado a busca por preditores de complicações graves e de escores para identificação de casos de maior dificuldade.^18 - 21^ A análise de fatores de risco para complicações intraoperatórias maiores, realizada no presente estudo, confirmou a importância do tempo de implante como preditor de complicações intraoperatórias. Além disso, identificou que a necessidade de mudança de estratégia no transcorrer do procedimento também esteve associada a este tipo de problema. Este conhecimento pode ser de grande valia para a tomada de decisão intraoperatória, dando ao cirurgião a possibilidade de interromper o procedimento nos casos em que a extração é opcional ou de converter para técnica aberta sob CEC nos casos de infecção, antes que uma complicação catastrófica ocorra.

A mortalidade total elevada nos primeiros 30 dias após procedimentos de extração de cabos-eletrodos é motivo de preocupação, justificando, inclusive a criação de um nomograma para predição do risco de morte.^18^ Fatores não modificáveis e inerentes ao próprio paciente têm sido as principais causas de morte, tais como: idade avançada, insuficiência renal crônica ou a própria infecção no DCEI.^18^ O presente estudo confirmou que a presença de insuficiência renal, a classe funcional avançada para insuficiência cardíaca e a presença de infecção no DCEI são preditores independentes de morte aos 30 dias. Fatores relacionados à operação, como a necessidade de mais de um procedimento para a extração e a ocorrência de complicações maiores intraoperatórias, também foram preditores de mortalidade. A identificação da presença de cabo-eletrodo de CDI como fator de proteção chamou nossa a atenção, por caminhar no sentido oposto do que tem mostrado a literatura. A observação detalhada da população do presente estudo, entretanto, explicou esse achado: os portadores de CDI eram, de maneira geral, 10 anos mais jovens e apresentavam menor taxa de infecção no DCEI, fatores que podem ter agido como confundidores.

Embora a amostra do estudo seja bastante representativa, ela reflete as práticas assistenciais de um único hospital, considerado um centro de referência em extração transvenosa de cabos-eletrodos. Dessa forma, os resultados podem ter sido influenciados pelo nível de experiência da equipe cirúrgica e pela disponibilidade de infraestrutura específica para esse tipo de procedimento.

Em virtude da falta da incorporação da extração transvenosa por técnicas especiais no rol de procedimentos do SUS, o presente estudo não foi projetado para comparar os resultados das diferentes técnicas de extração, uma vez que a escolha da ferramenta foi definida por sua disponibilidade para cada caso. Esse tipo de comparação será realizado em futuros estudos conduzidos na nossa instituição em parceria com centros de outras localidades do Brasil.

## Conclusões

Nosso estudo demonstrou que a extração de cabos-eletrodos é um tratamento efetivo e seguro, com 1,4% de complicações diretamente associadas ao procedimento. A mortalidade expressiva de 9,3%, no período de observação do estudo, foi decorrente, principalmente, de complicações infecciosas prévias, relacionadas à própria indicação para o procedimento de extração. Foram identificados fatores de risco inerentes ao paciente e ao procedimento cirúrgico, o que permitirá o estabelecimento de estratégias preventivas nos pacientes com maior risco de apresentar eventos desfavoráveis.
