# Supplementary material for: Efetividade e Segurança da Remoção de Cabos-Eletrodos Transvenosos de Marca-Passos e Desfibriladores Implantáveis no Cenário da Prática Clínica Real
Source: Arq Bras Cardiol. 2020 Dec 1;115(6):1114–24. [Article in Portuguese] doi: 10.36660/abc.20200476 (PMC8133723; doi:10.36660/abc.20200476)
Supplement: Translation

# Translation: Effectiveness and Safety of Transvenous Removal of Cardiac Pacing and Implantable Cardioverter-defibrillator Leads in the Real Clinical Scenario

**Authors:** Roberto Costa, Katia Regina da Silva, Elizabeth Sartori Crevelari, Wagner Tadeu Jurevicius Nascimento, Marcia Mitie Nagumo, Martino Martinelli, Fabio Biscegli Jatene

**Affiliations:** 1 Brazil Instituto do Coração do Hospital das Clínicas da Faculdade de Medicina da Universidade de São Paulo, São Paulo, SP – Brazil

**Keywords:** Electrodes Implanted, Pacemaker, Cardiac Pacing Artificial, Transvenous lead extracion, Infection, Effectiveness, Surgical complications, Mortality

## Abstract

**Background:**

Transvenous lead extraction (TLE) of cardiac implantable electronic devices (CIED) is an uncommon procedure and requires specialized personnel and adequate facilities.

**Objectives:**

To evaluate the effectiveness and safety of the removal of CIED leads and to determine risk factors for surgical complications and mortality in 30 days.

**Methods:**

Prospective study with data derived from clinical practice. From January 2014 to April 2020, we included 365 consecutive patients who underwent TLE, regardless of the indication and surgical technique used. The primary outcomes were: success rate of the procedure, combined rate of major complications and intraoperative death. Secondary outcomes were: risk factors for major intraoperative complications and death within 30 days. Univariate and multivariate analysis were used, with a significance level of 5%.

**Results:**

Procedure success rate was 96.7%, with 90.1% of complete success and 6.6% of clinical success. Major intraoperative complications occurred in 15 (4.1%) patients. Predictors of major complications were: lead dwelling time ≥ 7 years (OR = 3.78, p = 0.046) and change in surgical strategy (OR = 5.30, p = 0.023). Functional class III-IV (OR = 6.98, p <0.001), renal failure (OR = 5.75, p = 0.001), CIED infection (OR = 13.30, p <0.001), number of procedures performed (OR = 77.32, p <0.001) and major intraoperative complications (OR = 38.84, p <0.001) were predictors of 30-day mortality.

**Conclusions:**

The results of this study, which is the largest prospective registry of consecutive TLE procedures in Latin America, confirm the safety and effectiveness of this procedure in the context of real clinical practice. (Arq Bras Cardiol. 2020; 115(6):1114-1124)

## Introduction

Cardiac implantable electronic device (CIED) lead extraction is an uncommon procedure, which requires long-term professional training and adequate facilities.^1 - 5^Although its indications are well established in medical guidelines, its use varies according with the expertise of each center, being almost exclusively used for the treatment of CIED-related infections in low volume centers and on a larger scale in centers with greater experience.^2 - 9^

Since most implantable devices use the venous access for lead placement, the transvenous techniques are the most commonly used. Nowadays, opening the chest is not frequently used in lead extraction, being almost exclusively limited to epicardial lead placement. For transvenous leads, the open surgery is restricted to cases where large lead vegetations are found or for correction of complications during transvenous lead extraction.^3^

Lead adhesion to the myocardium, to the veins or lead-to-lead adhesion are quite frequent. Several factors are related with adhesion formation, including: dwelling time of implanted leads, lead type, the number of leads, the patient’s age and sex.^6^In order to disrupt these adhesions, specific tools have been employed, each with its indications, advantages and disadvantages.^3 , 7^In general, the reported success rates for extractions range from 90 to 98%.^1 , 7 , 10 , 11^In spite of the satisfactory results obtained, catastrophic complications may occur during extraction, and when they do occur, open heart surgical procedures may be required for correction.^9 , 12 , 13^Severe complications are reported in 1 to 10% and intraoperative death, in 0.2 to 5.7%. The overall mortality rate within 30 days of surgery ranges from 2.1 to 10% of cases.^10 - 13^

The effectiveness of the tools used for extraction and the risk factors associated with the occurrence of catastrophic complications are not well established in the literature.^1 , 2 , 10 - 14^The low rate of utilization and complications, combined with the diversity of approaches and tools available for extraction, make comparison difficult. In the national setting, the lack of incorporation of specific lead extraction technology in the Brazilian Unified Health System (SUS) explains its limited use, as well as the absence of reliable data resulting from lead extraction in Brazil.

The purpose of this study was to assess the effectiveness and the safety of pacemaker and implantable defibrillator lead extraction, aiming at determining the procedures’ success rate, the rate of surgical complications, surgical death rate and total mortality within 30 days after hospital discharge, as well as the risk factors for unfavorable outcomes.

## Methods

### Study design and setting

This is a prospective observational study with data derived from clinical practice. The data were collected at two distinct moments: (1) on index hospital admission, i.e., the episode of hospital care related to the lead extraction procedure; (2) thirty days after hospital discharge.

This study was performed in a high-complexity cardiology hospital, and was approved by the institution’s Research Ethics Committee.

### Study Population

From January 2014 to April 2020, all the patients submitted to lead extraction were consecutively included, regardless of surgical indication and the technique used. Individuals who were submitted to lead extraction due to orthotopic cardiac transplantation were excluded.

### Study outcomes

The primary outcomes of the study were the procedure effectiveness, expressed by the clinical procedural success, and its safety, expressed by the combined rate of major complications and intraoperative mortality.

The definitions of European guidelines were used.^8^In this study, clinical procedural success was defined as the removal of all targeted leads and material or a retention of <4 cm of the lead, as long as the extraction was not performed for the treatment of CIED infections. On the other hand, procedural failure was defined as the retention of more than 4 cm of lead material; the retention of lead material of any size in patients with CIED infection; or the development of any permanently disabling complication or procedural-related death.

The secondary outcomes were: risk factors for major intraoperative complications and death within 30 days after discharge.

### Study phases

#### Preoperative evaluation

Patients with indication for lead extraction and who fulfilled the study eligibility criteria underwent routine preoperative evaluation, including clinical evaluation, laboratory and imaging tests.

Following the institution’s routine protocol, the patients were submitted to chest X-ray, to determine lead position; two-sided digital venography to investigate the venous territory and, when there was the diagnosis of CIED infection, transesophageal echocardiogram.

#### Surgical Procedure

The procedures were performed under general anesthesia with orotracheal intubation, full monitoring, including transesophageal echocardiogram.

The surgeries were grouped according to the lead access route: (1) Epicardial leads, extracted by open surgery; (2) Transvenous leads, extracted using preferably transvenous techniques.

#### Lead removal using the venous entry site approach

Leads that allowed for extravascular handling were removed using a sequential approach, which was initiated with a simple direct traction and moved towards transvenous extraction, with the use of specific tools, if direct traction was not successful.

- Removal by simple traction: an attempt to remove the leads with no specific extraction tools was made in all cases. To achieve this purpose, a standard stylet was passed through the lumen of the lead to be removed, with firm, continuous traction, in order to separate the lead from the myocardium and venous system. In case this approachwas not successful, the following step was the use of extraction devices.
- Transvenous extraction: the extraction procedure was initiated with the use of a locking stylet. The intravascular dissection was performed with the extraction tools available for the case (laser-powered sheath, mechanical sheath or rotational mechanical sheath). The purpose of the dissection was to guide the sheath until the lead placement site in the myocardium in order to perform the counter-traction.

#### Lead removal through intravascular capture

In cases where the lead was not accessible for extravascular manipulation (free-floating leads), intravascular capture was performed through femoral or jugular access. After intravascular capture, the lead tip was removed by simple traction or counter-traction, depending on the necessity of each case.

#### Change in Surgical Strategy

In the cases where the first-choice extraction tool cannot be advanced over the lead, a change in strategy may be necessary, including the use of another extraction tools, intravascular capture or open surgery.

#### CIED reimplantation

In the patients who were not diagnosed with CIED infection, the placement of the new device was performed during the same surgical procedure. When CIED infection was present, the new implantation was always performed in a separate surgery, after the infection was controlled.

## Postoperative assessment and clinical follow-up

At discharge, immediate postoperative data were collected, prioritizing the investigation of perioperative complications.

In accordance with normal routine, all patients were evaluated in the outpatient setting, 30 days after discharge. This evaluation prioritized the investigation of complications resulting from the procedure, the need for readmission or surgical reintervention.

## Electronic data collection and management

To ensure database quality, a previously standadized infraestructure was adopted,^15^which included: (1) Data management planning; (2) Definition of data element terminology; (3) Development of electronic forms using the REDCap platform;^16^(4) Parameterization of specific functions of the REDCap; (5) Data collection team training; (6) Dynamic monitoring of database quality; (7) Integration of the REDCap system with the Business Intelligence tool, to create interactive dynamic dashboards, allowing for real-time result view, in a cloud technology environment. To favor the study’s reproducibility and publishing of anonymized and de-identified data in real time, we opted for the Open Source platform (Shinydashboard, RStudio) ( Figure1 ).

**Figura 1:** – Electronic data capture and study data management. A – Data management steps. B – REDCap’s functionalities used in the study.

## Variables studied and statistical analysis

The following were analyzed as independent variables for outcomes: demographic data, baseline clinical data, characteristics of the removed CIED, type of extraction and use of specific extraction tolls.

A univariate analysis was used to investigate the risk factors associated with the outcomes, adopting a level of significance of 5%. The multivariate logistic regression model was used with the stepwise method of variable selection for the investigation of independent risk factors, using as inclusion criteria the associations with a p-value ≤ 0.10 in the univariate analysis.

## Results

### Baseline characteristics

During the study period, we included 365 patients who had undergone one to three lead extraction procedures until treatment conclusion. There was a prevalence of male individuals (55.6%), and the average age was of 59.8 ± 19.3 years, with a median of 63.0 years ( Table 1 ).

**Table 1:** – Demographic and clinical baseline data

Baseline Characteristics	
Males, n (%)	203 (55.6)
Age (years). mean ± SD (variation)	59.8 ± 19.3 (0.8 - 98.0)
Caucasian. n (%)	306 (83.8)
**Functional Class (NYHA). n (%)**	
I – II	314 (86.0)
III - IV	51 (14.0)
**Structural heart disease. n (%)**	
Conduction and rhythm disorders (without structural heart disease)	153 (41.9)
Chagas’ Disease	68 (18.6)
Ischemic cardiomyopathy	46 (12.6)
Non-ischemic cardiomyopathy	86 (23.6)
Congenital cardiac defect	12 (3.3)
**Comorbidities. n (%)**	
Hypertension	186 (51.0)
Diabetes	60 (16.4)
Atrial Fibbrilation	70 (19.2)
Heart Failure	140 (38.4)
Chronic kidney failure	43 (11.8)
Stroke	33 (9.0)
Neoplasia under current or recent treatment	9 (2.5)
Left ventricular ejection fraction. mean ± SD (variation)	49.1 ± 16.3 (15- 82.0)
**Type of CIED in use. n (%)**	
Unicameral PM	41 (11.2)
Dual chamber PM	170 (46.6)
Unicameral ICD	33 (9.0)
Dual chamber ICD	64 (17.5)
CRT-PM	24 (6.6)
CRT-ICD	33 (9.0)
**Indication for lead extraction. n (%)**	
Infection treatment	104 (28.5)
Multiples abandoned leads	207 (56.7)
Obtaining venous access	22 (6.0)
Lead dislodgement	26 (7.1)
Treatment of thromboembolic complications	3 (0.8)
Other conditions	3 (0.8)
**Main diagnosis. n (%)**	
Pulse generator pocket infection	57 (15.6)
Intravascular infection without vegetation	10 (2.7)
Intravascular infection with vegetation	37 (10.1)
Lead Dysfunction	218 (59.7)
Indication of upgrade procedure	35 (9.6)
Others	8 (2.2)

*ICD: Implantable Cardioverter-Defibrillator; SD: standard deviation; PM: pacemaker; NYHA: New York Heart Association; CRT: cardiac resynchronization therapy; CIED: Cardiac implantable electronic device.*

Most individuals were oligosymptomatic for heart failure (86.0%), with absence of structural cardiac disease in 39.1% of them. The devices previously implanted were pacemakers in 57.8% and implantable cardioverter-defibrillators (ICD) in 33.1% of the cases.

The main reason for the procedure was lead dysfunction, in 218 (59.7%) patients. CIED infection, with or without intracardiac vegetations, was the cause for removal in 104 (28.5%) of the cases.

### Surgical Characteristics

A total of 378 lead extraction procedures were performed in 365 patients. In 9 (2.5%) cases more than one procedure was required. A total of 634 leads, 521 pacemakers and 113 ICDs were removed. The mean lead dwelling time was 7.5 ± 6.6 years with a maximum of 39 years ( Table 2 ).

**Table 2:** – Lead Extraction Procedure Data

Surgical Characteristics	
Procedure duration (minutes). mean ± SD (variation)	147.9 ± 82.0 (20 - 635)
**Total number of leads extracted per patient. n (%)**	
One	197 (54.0)
Two	125 (34.2)
Three	32 (8.8)
Four	11 (3.0)
Lead dwelling time (years). mean ± SD (variation)	7.5 ± 6.6 (0 – 39)
**Lead implantation site. n (%)**	
Atrial	221 (34.9)
Right ventricle (pacemaker)	258 (40.7)
Right ventricle (ICD)	113 (17.8)
Coronary sinus	42 (6.6)
**Main Surgical Technique. n (%)**	
Simple traction	140 (38.4)
Simple traction with dilation	22 (6.0)
Transvenous lead extraction by a venous entry site approach	180 (49.3)
Thoracotomy without cardiopulmonary bypass	17 (4.7)
Thoracotomy with cardiopulmonary bypass	6 (1.6)
**Specific Extraction Tools. n (%)**	
Locking stylet	183 (50.1)
Mechanical sheath	77 (21.1)
Rotational mechanical sheath	23 (6.3)
Laser-powered sheath	80 (21.9)
**Change in Surgical Strategy. n (%)**	
Simple traction removal to transvenous extraction	2 (0.5)
Simple traction removal to open surgery	2 (0.5)
Change in the type of intravascular dissection tool	6 (1.6)
Venous entry site extraction approach to intravascular capture	5 (1.4)
Venous entry site extraction approach to open surgery	5 (1.4)
Multiple	1 (0.3)

*ICD: implantable cardioverter defibrillator; SD: standard deviation.*

Open surgery for epicardial lead extraction was performed in 17(4.6%) patients. Surgery with Cardiopulmonary Bypass (CPB) was required as first approach in 6 (1.6%) and, after as a second approach in 7 (1.9%) cases. In all procedures performed through venous entry, the simple traction was attempted, which was successful in 140 (38.4%) patients. In the other patients, transvenous extraction was performed. A locking stylet was used in 183 cases, laser-powered sheath in 80 (21.9%), mechanical sheath in 77 (21.1%) and rotational mechanical sheath in 23 (6.3%) patients.

A change in strategy was required in the same surgical procedure in 12 (3.3%) patients and, as a separate procedure, in 9 (2.5%). The change in strategy demanded the use of another type of dissection tool in 6 (1.6%), intravascular capture in 5 (1.4%), or open surgery with CPB in 7 (1.9%) cases.

### Study Primary Outcomes

#### Procedure Effectiveness

Procedural success rate was of 96.7% (CI 95%= 94.5 – 98.5%), with complete procedural success in 329 (90.1%, CI 95%= 87.1 – 93.2%) patients and clinical procedural success in 24 (6.6%, CI 95%= 4.1 – 9.1%).

Procedural failure rate was of 3.2% (CI 95%= 1.5 – 5.1%). Failure was due to the retention of a fragment >4cm in 3 (0.8%) patients without infection, retention of any fragment in 7 (1.9%) patients with CIED infection and major complication that required surgical repair in 2 (0.5%) patients.

#### Procedure Safety

The composite outcome of major complications and intraoperative death occurred in 15 (4.1%, CI 95%= 2.1 – 6.1) patients. Intraoperative death occurred in only 1 (0.3%) case due to avulsion of cardiac structures, causing hemorrhagic shock. Only 2 (0.5%) patients had more than one major complication simultaneously. Minor intraoperative complications were observed in 10 (2.7%) patients, but in only one case there were concomitant minor complications ( Table 3 ).

**Table 3:** – Major and minor intraoperative lead extraction-related complications

Intraoperative complications	
Major complications. n (%)	
Death	1 (0.3)
Cardiorespiratory arrest	6 (1.6)
Unstable arrhythmia	5 (1.4)
Cardiac tamponade	3 (0.8)
Avulsion of cardiac structures	2 (0.5)
Unstable bleeding	3 (0.8)
**Minor complications. n (%)**	
Hemothorax requiring drainage	2 (0.5)
Pneumothorax requiring drainage	2 (0.5)
Minimal pneumothorax.	1 (0.3)
Pericardial effusion	4 (1.1)
Unstable bleeding	4 (1.1)
Lead dislodgement	2 (0.5)

#### Postoperative events

The patients’ median hospital length of stay was 9 days, ranging from 1 to 169 days. The main reason for long hospital stay was the prolonged antibiotic therapy required by patients with CIED infection. After discharge, 13 (3.6%) patients had complications and 8 (2.2%) were submitted to new surgery ( Table 4 ).

**Table 4:** – Postoperative events after lead extraction

Postoperative events	
Length of hospital stay (days). mean ± SD (variation)	17.4 ± 21.6 (1- 169)
**In-hospital major complications**	
Death	29 (7.9)
Cardiorespiratory arrest	3 (0.8)
Unstable arrhythmia	1 (0.3)
Cardiac tamponade	1 (0.3)
Pulmonary embolism	2 (0.5)
Unstable bleeding	2 (0.5)
Sepsis	13 (3.6)
**In-hospital minor complications**	
Hemothorax requiring drainage	1 (0.3)
Pneumothorax requiring drainage	2 (0.5)
Pocket hematoma	6 (1.6)
Lead dislodgement	2 (0.5)
Complications within 30 days after **discharge**	
Death	4 (1.1)
Readmission	33 (9.0)
Device-related reoperation	8 (2.2)
CIED-related infection	1 (0.3)
Lead dysfunction	3 (0.8)
Pulmonary embolism	2 (0.5)
Deep vein thrombosis of the upper extremity	7 (1.9)

*SD: standard deviation; CIED: Cardiac implantable electronic device.*

The total mortality was of 34 (9.3%) cases: 1 (0.3%) intraoperative death, 29 (7.9%) in the in-hospital postoperative period and 4 (1.1%) after discharge. The most frequent cause of mortality was CIED infection, in 20 (5.5%) cases, followed by cardiovascular-related causes in 6 (1.6%), extraction procedure complications in 5 (1.4%) and non-cardiovascular causes in 3 (0.8%) patients.

#### Risk factors for major intraoperative complications and mortality

According with the univariate analysis, the lead dwelling time (p= 0.015) and change in surgical strategy (p=0.016) were associated with higher occurrence of major intraoperative complications.

Age (p= 0.004), functional class (p<0.001), heart failure (p= 0.003), renal failure (p<0.001), CIED-related infection (p<0.001), total number of removed leads (p<0.001), type of lead removed (p=0.029), total number of procedures (p<0.001), procedure results (p=0.002), change in surgical strategy (p= 0.005) and major intraoperative complications (p<0.001) were all factors associated with total mortality within 30 days ( Table 5 ).

**Table 5:** – Risk factors for major intraoperative complications and total mortality within 30 days

Characteristics	Complications (absent) (n= 350)	Complications (presente) (n= 15)	p-value	Death (absent) (n= 331)	Death (present) (n= 34)	p-value
Male sex. n (%)	197 (56.3)	9 (60.0)	0.214	185 (55.9)	18 (52.9)	0.741
Age (years). mean ± SD	59.9 ± 19.2	57.7 ± 22.5	0.670	58.8 ± 19.4	69.0 ± 15.9	0.004
**Functional Class (NYHA). n (%)**						
I – II	303 (86.6)	11 (73.4)	0.142	292 (88.2)	22 (64.7)	
III – IV	47 (13.4)	4 (26.6)		39 (11.8)	12 (35.3)	<0.001
**Cardiac Disease. n (%)**						
Conduction and rhythm disorders	147 (42.0)	6 (40.0)		142 (42.9)	11 (32.3)	
Ischemic cardiomyopathy	46 (13.1)	0	0.258	42 (12.7)	4 (11.8)	0.420
Non-Ischemic cardiomyopathy	157 (44.9)	9 (60.0)		147 (44.4)	19 (55.9)	
**Comorbidities. n (%)**						
Diabetes (absent)	290 (82.9)	15 (100)		278 (84.0)	27 (79.4)	
Diabetes (present)	60 (17.1)	0	0.145	53 (16.0)	7 (20.6)	0.493
Heart failure (absent)	217 (62.0)	8 (53.3)		212 (64.1)	13 (38.2)	
Heart failure (present)	133 (38.0)	7 (46.7)	0.499	119 (35.9)	21 (61.8)	0.003
Chronic kidney failure (absent)	307 (87.7)	15 (100)		299 (90.3)	23 (67.6)	
Chronic kidney failure (present)	43 (12.3)	0	0.233	32 (9.7)	11 (32.4)	<0.001
**CIED type. n (%)**						
Unicameral	71 (20.3)	3 (20.0)		66 (19.9)	8 (23.5)	
Dual chamber	225 (64.3)	9 (60.0)	0.862	216 (65.3)	18 (53.0)	0.296
Cardiac resynchronization therapy	54 (15.4)	3 (20.0)		49 (14.8)	8 (23.5)	
**Indication for extraction. n (%)**						
Non-infectious causes	250 (71.4)	11 (73.3)		253 (76.4)	8 (23.5)	
CIED infection	100 (28.6)	4 (26.7)	1.000	78 (23.6)	26 (76.5)	<0.001
**Number of leads removed. n (%)**						
1 - 2	310 (88.6)	12 (80.0)		301 (90.9)	21 (61.8)	
3 - 4	40 (11.4)	3 (20.0)	0.400	30 (9.1)	13 (38.2)	<0.001
Lead dwelling time. n (%)	7.3 ± 6.6	11.2 ± 6.7	0.015	7.1 ± 6.2	10.1 ± 9.5	0.142
**Lead type. n (%)**						
Pacemaker	252 (72.0)	12 (80.0)		234 (70.7)	30 (8.2)	
ICD	98 (28.0)	3 (20.0)	0.768	97 (29.3)	4 (11.8)	0.029
**Surgical technique. n (%)**						
Simple traction with or without dilation	159 (45.4)	3 (20.0)		148 (44.7)	14 (41.2)	
Transvenous extraction	169 (48.3)	11 (73.3)	0.140	164 (49.6)	16 (47.1)	0.386
Open surgery	22 (6.3)	1 (6.7)		19 (5.7)	4 (11.7)	
**Change in surgical strategy. n (%)**						
No	339 (96.8)	12 (80.0)		322 (97.3)	29 (85.3)	
Yes	11 (3.2)	3 (30.0)	0.016	9 (2.7)	5 (14.7)	0.005
**Number of procedures performed. n (%)**						
One	342 (97.7)	14 (93.3)		328 (99.1)	28 (82.4)	
Two	5 (1.4)	1 (6.7)	0.317	3 (0.9)	3 (8.8)	<0.001
Three	3 (0.9)	0		0	3 (8.8)	
**Procedure results. n (%)**						
Successful	340 (97.1)	13 (86.7)		324 (97.9)	29 (85.3)	
Failure	10 (2.9)	2 (13.3)	0.082	7 (2.1)	5 (14.7)	0.002
**Major intraoperative complications. n (%)**						
Absent	-	-		323 (97.6)	27 (79.4)	
Present	-	-		8 (2.4)	7 (20.6)	<0.001

*SD: standard deviation; CIED: Cardiac implantable electronic device.*

Using multivariate analysis, it was possible to identify that lead dwelling time ≥ 7 years and change in surgical strategy as independent predictor factors for the occurrence of major intraoperative complications. Functional classes III-IV, chronic kidney failure, CIED infection, number of procedures performed and major intraoperative complications were independent factors for total mortality within 30 days ( Table 6 ).

**Table 6:** – Independent predictors of intraoperative major complications and total mortality

Independent predictors	Odds ratio	CI 95%		p-value
Intraoperative major complications				
Lead dwelling time ≥ 7 years	3.78	1.02	13.95	0.046
Change in surgical strategy	5.30	1.26	22.22	0.023
**Mortalility**				
Functional Class (NYHA) III - IV	6.98	2.45	19.86	<0.001
Chronic Kidney Failure	5.75	1.98	16.67	0.001
CIED infection	13.30	4.45	39.69	<0.001
Total number of procedures	77.32	8.64	692.19	<0.001
Major intraoperative complications	38.84	7.83	192.77	<0.001
ICD lead extraction	0.22	0.06	0.81	0.023

*CIED: Cardiac implantable electronic device.*

## Discussion

This study is the first registry in Latin America designed to assess, prospectively, systematized data of lead extraction procedures in real clinical practice setting. Therefore, this sample composed of 634 removed leads from 365 patients, followed during 30 days after discharge, is representative of patients of all ages, with different structural cardiomyopathies and comorbidities, as well as all types of CIED and leads.

The finding that CIED infection was not the main indication for lead extraction differs from most studies, in which more than half of the population suffers from infection.^3 , 7 , 11 , 12^The high prevalence of non-infected individuals was essential for the achievement of the study objectives, since they are younger patients, with long lead implant duration. These characteristics increased the representativeness of complications associated with the procedure itself and its impact on mortality, at some extent minimizing the effects of infection, comorbidities and other characteristics inherent to the patient.

The development of specific tools for transvenous extraction has been essential to ensure greater patient safety. The comparison of safety and effectiveness of these devices, however, is problematic, because many of them are used as backup solutions for difficult cases or for correcting complications.^8 , 12 - 14^In this study, the main types of tools available for transvenous extraction were used. The lack of incorporation of transvenous extraction by the SUS, however, prevented an adequate comparison between the several technologies available, since the choice of device was defined by its availability for each case. Despite this bias, a locking stylet was used in 50.1% of the cases, laser-powered sheaths in 21.9%, mechanical sheaths in 21.1%, and rotational mechanical sheaths in 6.3% of patients.

Thus, safety and effectiveness of lead extraction, the study primary outcomes, could be robustly assessed. The success rate of 96.7%, as well as the major complication rate of 4.1% were comparable to those of international services, with a large volume of extractions.^7 , 12 , 14 , 17 , 18^The total mortality rate of 9.3% was mainly a result of patient-related causes and, in only 1.4%, due to extraction-related complications.

The risk of catastrophic complications has been the main obstacle for the indication of lead extraction in optional cases. This fact has motivated the search for predictors of severe complications and scores for the identification of more difficult cases.^18 - 21^The risk factor analysis for major intraoperative complications performed in this study corroborated the importance of time of implantation as a predictor of intraoperative complications. In addition, it indicates that the need to change the strategy during the procedure has also been associated with this type of problem. This knowledge can provide valuable contributions for intraoperative decision-making, giving the surgery team the possibility of interrupting the procedure in cases where the extraction is optional or moving into an open technique with CPB in cases of infection, before a catastrophic complication occurs.

The high total mortality within 30 days after lead extraction procedures is a reason of concern, and may even justify the construction of nomograms to predict the risk of death.^18^Non-modifiable risk factors, inherent to the patient, have been the main causes of death, such as: advanced age, chronic kidney failure or the CIED infection itself.^18^This study confirmed that the presence of renal failure, the advanced functional class for heart failure and the presence of CIED infection are independent mortality predictors within 30 days. Procedure related factors, such as the need of another procedure for the extraction and the occurrence of major intraoperative complications were also mortality predictors. The identification of the presence of ICD lead as a protective factor called our attention, because it contradicts what has been shown in the literature. The detailed observation of the study population, however, explained this findind: ICD patients were, in general, 10 years younger, with lower rates of device infection, which may have been a confounding factor.

Although the study sample is quite representative, it reflects the care practices of a single hospital, considered a reference center in transvenous lead extraction. Therefore, the results may have been influenced by the surgical staff experience level and by the specific infrastructure available for this type of procedure.

Due to the lack of incorporation of transvenous extraction using special techniques in the list of procedures performed in our public health system, this study was not designed to compare the results of the different extraction techniques, since the choice of the extraction tool was determined by its availability for each case. This type of comparison will be made in future researches carried out at our institution in partnership with centers of other locations in Brazil.

## Conclusions

Our study showed that lead extraction is an effective and safe treatment, with 1.4% complications directly associated with the procedure. The expressive mortality rate, of 9.3% during the study observation, was a result, mainly, of prior infectious complications, related with the indication for the extraction procedure itself. Risk factors inherent to the patient and to the surgical procedure were identified, which will allow for the establishment of preventive strategies in the patients at a higher risk of presenting unfavorable events.
